# Identification of *DNAH17* Variants in Han-Chinese Patients With Left–Right Asymmetry Disorders

**DOI:** 10.3389/fgene.2022.862292

**Published:** 2022-05-27

**Authors:** Xuehui Yu, Lamei Yuan, Sheng Deng, Hong Xia, Xiaolong Tu, Xiong Deng, Xiangjun Huang, Xiao Cao, Hao Deng

**Affiliations:** ^1^ Health Management Center, The Third Xiangya Hospital, Central South University, Changsha, China; ^2^ Center for Experimental Medicine, The Third Xiangya Hospital, Central South University, Changsha, China; ^3^ Disease Genome Research Center, Central South University, Changsha, China; ^4^ Department of Neurology, The Third Xiangya Hospital, Central South University, Changsha, China; ^5^ Department of Pharmacy, Xiangya Hospital, Central South University, Changsha, China; ^6^ Department of Emergency, The Third Xiangya Hospital, Central South University, Changsha, China; ^7^ Department of General Surgery, The First Affiliated Hospital of Hunan University of Chinese Medicine, Changsha, China

**Keywords:** *DNAH17*, left–right asymmetry disorders, whole-exome sequencing, gene variants, ciliary/flagellar disorders

## Abstract

The formation of left–right asymmetry of the visceral organs is a conserved feature of the human body, and the asymmetry specification of structure and function is precisely orchestrated by multiple regulatory mechanisms. The abnormal results of organ positioning situs arise from defective cilia structure or function during embryogenesis in humans. In this study, we recruited two unrelated Han-Chinese families with left–right asymmetry disorders. The combination of whole-exome sequencing and Sanger sequencing identified two compound heterozygous variants: c.4109C>T and c.9776C>T, and c.612C>G and c.8764C>T in the dynein axonemal heavy chain 17 gene (*DNAH17*) in two probands with left–right asymmetry disorders. We report for the first time a possible association between *DNAH17* gene variants and left–right asymmetry disorders, which is known as a causal gene for asthenozoospermia. Altogether, the findings of our study may enlarge the *DNAH17* gene variant spectrum in human left–right asymmetry disorders, pave a way to illustrate the potential pathogenesis of ciliary/flagellar disorders, and provide supplementary explanation for genetic counseling.

## Introduction

The visceral organs of vertebrates have a strikingly conserved left–right (LR) asymmetry of the organ situs that is manifested in the chest (heart and lungs) and abdomen (stomach, spleen, liver, intestine, and colon) (McGrath et al., 2003; Blum et al., 2014). The normal organ asymmetry present across the LR axis of the body is called situs solitus (SS) (Sung et al., 2016). It is well recognized that the leftward flow of extracellular fluid at the node (i.e., nodal flow) plays a major role in normal LR axis determination during embryogenesis (McGrath et al., 2003; Pennekamp et al., 2015). Human LR asymmetry disorders have an estimated probability of more than 1 in 8000 live births and can be divided into two broad classes: situs inversus totalis (SIT) and situs ambiguous (SA). SIT is a malformation featuring a complete mirror image reversal of the organs and is usually not related to major influence on the patient’s health (Levin, 2004; Sung et al., 2016). In contrast, SA, also termed heterotaxy, is defined as any abnormal organ display that was not SS or SIT and is highly associated with human congenital heart disease (CHD) (Zhu et al., 2006; Best et al., 2019; Chen et al., 2020).

Abnormalities in the typical development of laterality usually occur as a result of genetic lesions, which form a number of human heritable disorders with significant clinical implications, including primary ciliary dyskinesia (PCD), nephronophthisis, Carpenter syndrome 2, and male infertility (Olbrich et al., 2002; Otto et al., 2003; Levin, 2004; Twigg et al., 2012; Ding et al., 2020). Variations in the genes related to the development and function of nodal cilia often lead to human laterality defects. Up to now, more than 82 genes have been reported to be related to LR asymmetry disorders, including cilia- and flagella-associated protein family members, coiled-coil domain-containing family members, dynein axonemal assembly factors, dynein axonemal light chains, dynein axonemal intermediate chains, and dynein axonemal heavy chains (DNAHs) (Osório et al., 2019; Al Mutairi et al., 2020; Bustamante-Marin et al., 2020a; Bustamante-Marin et al., 2020b; Cannarella et al., 2020; Chen et al., 2020; Cho et al., 2020; Ding et al., 2020; Heigwer et al., 2020; Sahabian et al., 2020; Sha et al., 2020a; Thomas et al., 2020; Wang et al., 2020; Yang and Qi, 2020; Abdelhamed et al., 2021; Derrick et al., 2021; Guo et al., 2021; Wang et al., 2021). Genes belonging to the DNAH family, such as *DNAH1* (OMIM 603332), *DNAH5* (OMIM 603335), *DNAH6* (OMIM 603336), *DNAH9* (OMIM 603330), and *DNAH11* (OMIM 603339), are reported to be closely associated with cilia and/or flagella beating (Fliegauf et al., 2005; Hornef et al., 2006; Pifferi et al., 2010; Li et al., 2016). Variations in the dynein axonemal heavy chain 17 gene (*DNAH17*, OMIM 610063), encoding a component of outer dynein arms (ODAs) in the ciliary axonemes, have been reported to be associated with only flagella destabilization and asthenozoospermia (Whitfield et al., 2019; Zhang et al., 2020). There are, however, comparatively fewer studies that have investigated *DNAH17* and multiple morphological abnormalities of the flagella and asthenozoospermia, perhaps limited by the number of LR asymmetry phenotype-associated patients; further research is needed.

In the present study, whole-exome sequencing (WES) combined with Sanger sequencing was used to identify the potential causal gene and variants in two Han-Chinese families with LR asymmetry disorders, and compound heterozygous variants (c.4109C>T and c.9776C>T; c.612C>G and c.8764C>T) in the *DNAH17* gene were discovered.

## Materials and Methods

### Participants and Clinical Data

A 31-year-old healthy male and two unrelated Han-Chinese families were enrolled from the Third Xiangya Hospital. Central South University, and the First Affiliated Hospital of Hunan University of Chinese Medicine, Changsha, China. Available medical histories and examinations of the two probands were obtained. The entire study was approved by the Institutional Review Board of the Third Xiangya Hospital, Central South University, Changsha, China, and conducted following the tenets of the Declaration of Helsinki. Written informed consents were collected from all the participants or legal guardians.

### DNA Extraction and WES

The standard phenol–chloroform extraction method was used to isolate genomic DNA (gDNA) from peripheral blood leucocytes (Yuan et al., 2015). The Qubit dsDNA HS Assay kit (Invitrogen, Thermo Fisher Scientific, Inc.) was used to quantify the gDNA samples. WES for the probands of the two pedigrees was performed by the BGI-Shenzhen, China, as previously described (Zheng et al., 2016; Hu et al., 2017). The qualified gDNA samples were randomly fragmented by using Covaris E220 (Covaris, Inc.), and 150-250 bp fragments were selected using the Agencourt AMpure XP Kit (Beckman Coulter, Inc.). After the process of end-repairing, A-tailing reactions, and adaptor ligation, the DNA fragments were amplified *via* ligation-mediated PCR. The obtained products were purified and hybridized to the exome array for enrichment. The exome capture is based on the Agilent SureSelect Human All Exon V6 platform, which covers about 99% of the human exonic regions. Captured fragments were then circularized, and DNA nanoballs were produced by rolling circle amplification, which were loaded on BGISEQ-500 sequencing platforms (BGI-Shenzhen, China), according to the quality control standards and operation procedures (Huang et al., 2017).

### Read Mapping and Variant Analysis

After the process of the raw data filtering, the clean reads were mapped to the human reference genome (GRCh37/hg19) *via* the Burrows–Wheeler Aligner (BWA, v0.7.15) program (Li and Durbin, 2010). To make assurance of variant accuracy, local realignment and base quality recalibration were performed by using the genome analysis toolkit (GATK, v3.3.0, https://www.broadinstitute.org/gatk/guide/best-practices), following the removal of duplicate reads using Picard tools (v2.5.0, https://broadinstitute.github.io/picard/) (Van der Auwera et al., 2013). For the qualified data, strict quality control was guaranteed. HaplotypeCaller of GATK was used to call insertions and deletions (indels) and single nucleotide polymorphisms (SNPs). Next, SnpEff software (https://pcingola.github.io/SnpEff/) provided the variants with annotation. The annotation data and final variants were prepared for the downstream analysis (Pereira et al., 2020). All candidate variants were filtered against several public databases: the Single Nucleotide Polymorphism database (version 154, dbSNP154), National Heart, Lung and Blood Institute’s Exome Sequencing Project 6500 (NHLBI-ESP6500), 1000 Genomes Project (1000G), Exome Aggregation Consortium (ExAC), Genome Aggregation Database (gnomAD), and an in-house exome database of BGI-Shenzhen (Lim et al., 2013; Xia et al., 2017). Then, Sanger sequencing was applied to confirm the identified potential causal variants using an ABI 3500 sequencer (Applied Biosystems, Thermo Fisher Scientific, Inc.) (Guo et al., 2013; Xiao et al., 2018). Locus-specific polymerase chain reaction (PCR) amplification and sequencing primers were designed using the online Primer3 program (http://primer3.ut.ee/) and National Center for Biotechnology Information Basic Local Alignment Search Tool (NCBI BLAST, https://blast.ncbi.nlm.nih.gov/Blast.cgi) (Untergasser et al., 2012), and the paired primers are listed in Table 1.

**TABLE 1:** Detecting primers for the dynein axonemal heavy chain 17 gene variants.

Variant	Forward sequence (5′–3′)	Reverse sequence (5′–3′)	Product size (bp)
c.612C>G	GAT​CCC​CTC​TTC​ACT​GGA​CA	GAT​GCA​CTT​GAG​GTT​CAG​CA	184
c.4109C>T	CTC​GAC​AAC​ACC​GTG​AAA​AA	CAC​ATT​GGC​TTT​ACC​AGC​AT	228
c.8764C>T	TTA​TGG​AGG​ACG​AGG​TGG​AG	TCA​CAT​CCC​ATG​AAG​GAT​CA	239
c.9776C>T	GAG​TTC​ATC​CGC​TCC​AAG​TC	GGC​ACT​TAC​GGC​AAT​CTT​GT	186

### Bioinformatics Analyses

Several bioinformatic prediction software programs were used to estimate whether a variant is related to protein structure or function. For *in silico* analyses, Protein Variation Effect Analyzer (PROVEAN, http://provean.jcvi.org/index.php), Polymorphism Phenotyping version 2 (PolyPhen-2, http://genetics.bwh.harvard.edu/pph2/), and MutationTaster (https://www.mutationtaster.org/) were applied to get access to impacts on the protein structure and function (Adzhubei et al., 2010; Schwarz et al., 2014; Choi and Chan, 2015). NCBI BLAST was used to assess sequence conservation of the amino acid at variant positions among different species.

The protein structures of wild type and variant type were predicted *via* the online SWISS-MODEL tool (https://swissmodel.expasy.org/) and the visualized structures were further constructed *via* PyMOL software (version 2.3, Schrödinger, LLC, Portland, United States).

## Results

### Clinical Findings

**FIGURE 1:**
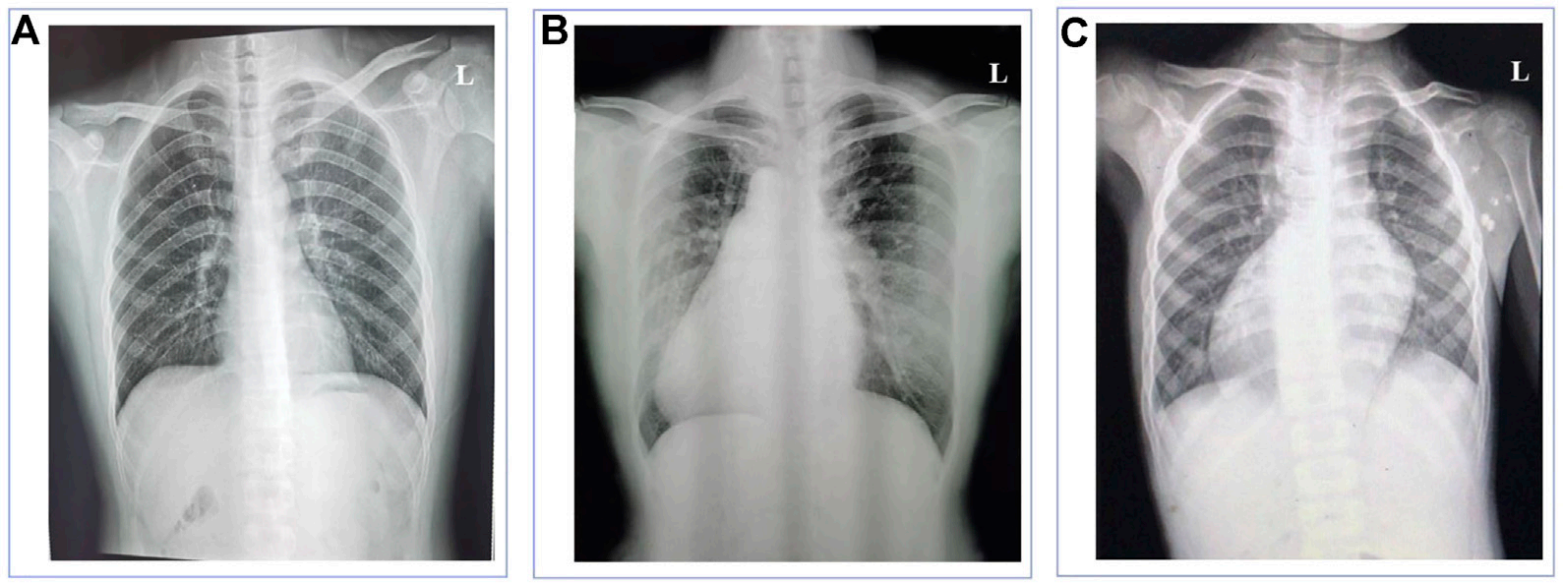
Chest X-ray images of the normal individual and patients with left-right asymmetry disorders. **(A)** Chest X-ray of the normal individual presented normal organ placement. **(B)** Chest X-ray of the proband in family 1 revealed dextrocardia. **(C)** Chest X-ray of the proband in family 2 revealed dextrocardia.

The normal individual presented normal organ placement (Figure 1A). Two probands from unrelated Han-Chinese families presented randomization of LR asymmetry. The proband 1 from family 1 is a 50-year-old woman whose chest X-ray and B-mode ultrasonographic diagnosis revealed the mirror image reversal of normal organ placement and no signs of other cilia-related disorders (Figure 1B). The proband 2 from family 2, a 5-year-old boy, was diagnosed with dextrocardia and complex CHD, including pulmonary valve stenosis, complete transposition of the great arteries, and endocardial cushion defect, by chest X-ray (Figure 1C), cardiac ultrasound, and CT scan. He was prone to having colds and coughs since early childhood. In addition, the available medical history showed that cardiac murmurs with cyanosis were discovered in infancy. The two probands declined further examinations such as transmission electron microscopy (TEM) and high-speed video microscopy (HSVM). Other members of the two families refused to participate in relative inspection, as they insisted on not suffering any cilia-related symptoms.

### Genetic Findings

**FIGURE 2:**
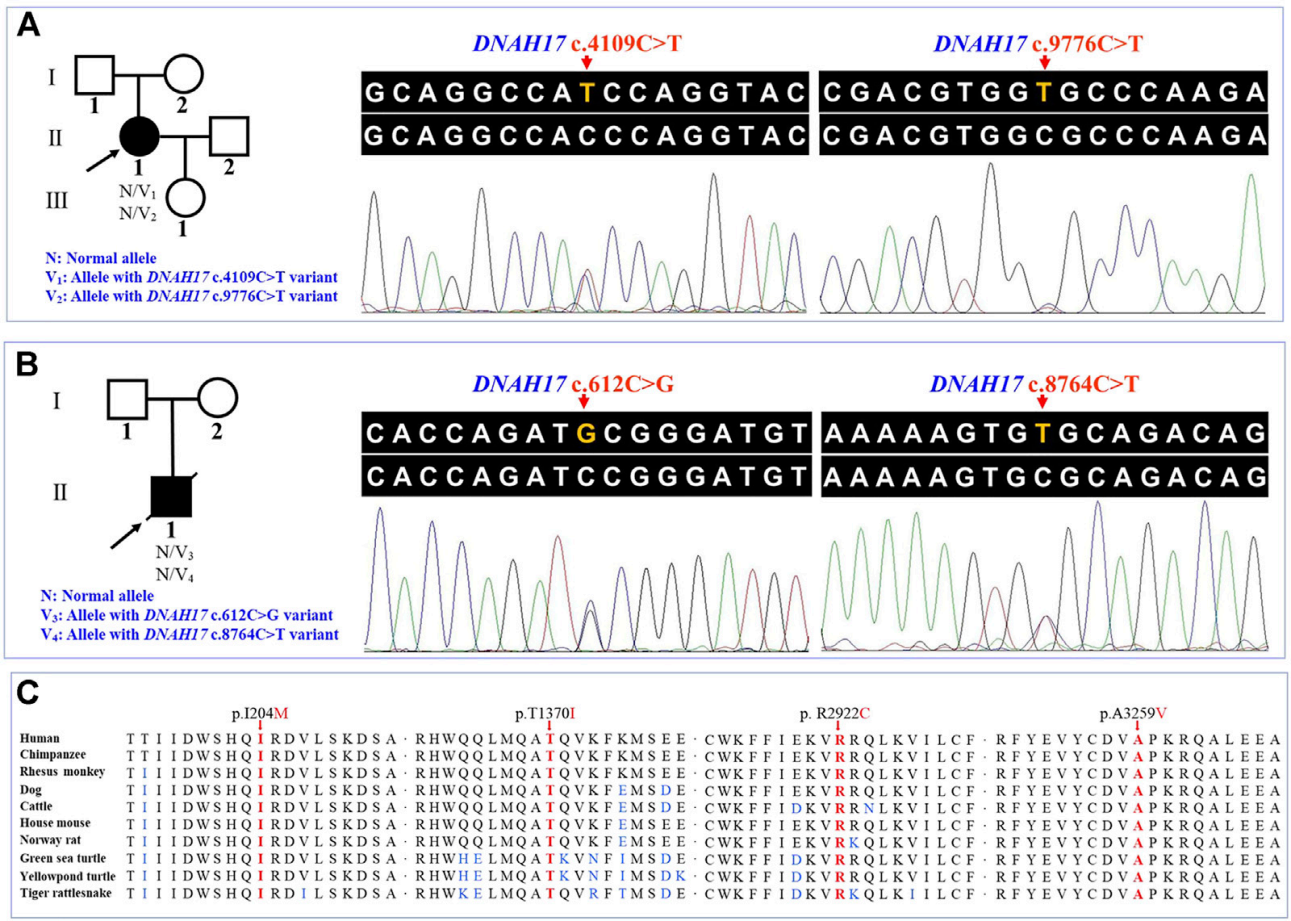
Pedigrees and sequence analysis of the two unrelated Han-Chinese patients with left-right asymmetry disorders. **(A)** and **(B)** Pedigrees with left-right asymmetry disorders and Sanger sequencing results. The proband, shown as a solid symbol, is indicated by an arrow, and the deceased family member is shown with a slash in the pedigree tree. **(C)** Sequence alignment of the dynein axonemal heavy chain 17 among different species, with the affected amino acids indicated by the arrows. *DNAH17*, the dynein axonemal heavy chain 17 gene.

WES of the proband 1 and the proband 2 generated a total of 242.17 million and 255.35 million clean reads with an average of 99.94% successfully mapped to the human reference genome (GRCh37/hg19). On the target region, the mean sequencing depth of 264.94-fold (proband 1) and 276.52-fold (proband 2) guaranteed enough accuracy to call variants in 99.63% and 99.71% of the targeted bases covered by at least 10×, respectively. There were a total of 105,991 SNPs and 18,461 indels detected in proband 1, while a total of 106,426 SNPs and 18,992 indels were detected in proband 2. A variant filtering strategy referring to previous studies was utilized to identify potential causal variants in these patients (Zheng et al., 2016; Xiang et al., 2019). The following were considered: (i) variants recorded in dbSNP154, NHLBI-ESP6500, and 1000G with minor allele frequency (MAF) ≥1% were ruled out. (ii) The remaining variants were further filtered again in the in-house BGI exome database with 1,943 Han-Chinese controls without randomization of LR asymmetry, and variants with MAF ≥1% were ruled out. (iii) Variants predicted to be deleterious were reserved. (iv) Compound heterozygous or homozygous variants in known genes responsible for LR asymmetry disorders or other cilia-related disorders were prosecuted as potential candidate variants. With these criteria, only two compound heterozygous variants: c.4109C>T and c.9776C>T, and c.612C>G and c.8764C>T in the *DNAH17* gene (NM_173628.4) were identified in two probands from unrelated families, respectively. Disease-causing variants in at least 82 of the known genes responsible for LR asymmetry disorder phenotypes were excluded in our patients, though gross deletion/duplication and complex rearrangement in these genes cannot be completely ruled out. These four variants are recorded in the dbSNP154 and has a low frequency in the global population of 1000G, ExAC, and gnomAD (Table 2 **)**, suggesting these two compound heterozygous variants are potential disorder-related variants. These four variants were further confirmed by Sanger sequencing (Figures 2A,B).

**TABLE 2:** *In silico* analysis of the dynein axonemal heavy chain 17 gene variants.

Nucleotide change	Amino acid change	dbSNP154	Variant type	Bioinformatics analysis				Allele frequencies		
				PROVEAN	SIFT	PolyPhen-2	MutationTaster	1000G	ExAC	gnomAD
c.612C>G	p.Ile204Met	rs577131115	Missense	Neutral	Tolerated	Possibly damaging	Polymorphism	2.00×10^-4^	1.17×10^-4^	5.91×10^-5^
c.4109C>T	p.Thr1370Ile	rs548985742	Missense	Deleterious	Tolerated	Possibly damaging	Disease causing	3.99×10^-4^	1.97×10^-4^	1.38×10^-4^
c.8764C>T	p.Arg2922Cys	rs367844100	Missense	Deleterious	Damaging	Probably damaging	Disease causing	2.00×10^-4^	-	-
c.9776C>T	p.Ala3259Val	rs151161879	Missense	Neutral	Damaging	Benign	Polymorphism	4.59×10^-3^	1.38×10^-3^	6.44×10^-4^

dbSNP154, Single Nucleotide Polymorphism database (version 154); rs, Reference SNP; PROVEAN, Protein Variation Effect Analyzer; SIFT, Sorting Intolerant from Tolerant; PolyPhen-2, Polymorphism Phenotyping version 2; 1000G, 1000 Genomes Project; ExAC, Exome Aggregation Consortium; gnomAD, Genome Aggregation Database.

### Variant Bioinformatics Analysis

**FIGURE 3:**
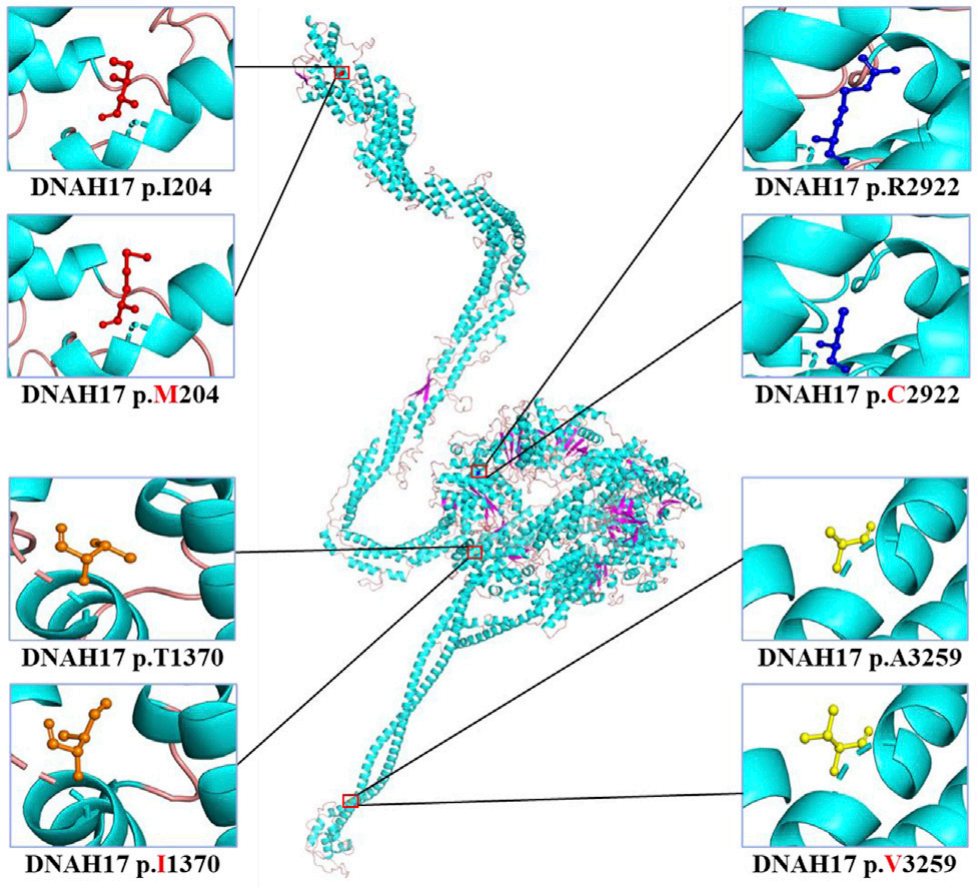
Cartoon model of the dynein axonemal heavy chain 17 protein structure visualized by PyMOL based on the SWISS-MODEL. The isoleucine (I) and mutated methionine (M) at position 204; threonine (T) and mutated isoleucine (I) at position 1370; arginine (R) and mutated cysteine (C) at position 2922; and alanine (A) and mutated valine (V) at position 3259 are indicated with ball-and-stick models.

The c.8764C>T (p.Arg2922Cys) variant was predicted to be “deleterious,” “damaging,” “probably damaging,” and “disease causing” by PROVEAN, Sorting Intolerant from Tolerant (SIFT), PolyPhen-2, and MutationTaster, respectively. For the other three variants, c.612C>G (p.Ile204Met), c.4109C>T (p.Thr1370Ile), and c.9776C>T (p.Ala3259Val), at least one of four prediction programs showed that the variants were potentially deleterious (Table 2 **)**. Alignment of the protein sequences across different species was shown by a phylogenetic analysis (Figure 2C), indicating that the variant sites were conserved in mammals and reptiles, further supporting that these variants are disorder-related variants. Structural modeling showed the conformational alteration in the context of protein (Figure 3).

## Discussion

The formation of LR asymmetry of the visceral organs is a conserved feature of human body, which is precisely orchestrated by multiple regulatory mechanisms (Blum et al., 2014; Sung et al., 2016; Hobbs et al., 2018). The process initiating in the node and occurring during embryogenesis can be divided into four steps: i) symmetry breaking caused by the leftward nodal flow, (ii) transmission of asymmetric signals to the left lateral plate mesoderm (LPM), (iii) cascades of Nodal and Lefty2 expression in the left LPM, and (iv) situs-specific morphogenesis (Ramsdell and Yost, 1998; Nonaka et al., 2005; Okada et al., 2005; Yoshimoto et al., 2012).

Rotational movement of motile monocilia in nodal cells creates the nodal flow and activates the asymmetric signaling, while the sperm flagella with similar structure are responsible for cell motility (McGrath et al., 2003; Shiraishi and Ichikawa, 2012; Pennekamp et al., 2015). Most motile cilia and sperm flagella share a highly conserved 9+2 axonemal structure (nine outer microtubule doublets surrounding one central microtubule pair), which are comprised of microtubules, motor dynein arms and the associated structures, exhibiting motile and sensory functions (Zhou et al., 2012; Ishikawa, 2017). Most immotile cilia have a 9+0 axoneme, lacking the central microtubule pair (Fliegauf et al., 2007). The inner and outer dynein arms (IDAs and ODAs), comprised of heavy, intermediate, and light dynein chains, are vital to motility of motile cilia and sperm flagella with 9+2 axonemes (King, 2016; Viswanadha et al., 2017; Lee and Ostrowski, 2021). Human LR asymmetry disorders are thought to be attributed to defective cilia structure or function during embryonic development (Blum et al., 2014; Shinohara and Hamada, 2017). Thus, exploring gene variants targeted establishment and function of nodal cilia during early embryogenesis may help the diagnosis and gene therapy of LR asymmetry disorder.

The *DNAH17* gene, located at chromosome 17q25.3, is a large gene composed of 81 exons and encodes an axonemal dynein heavy chain of ODA. DNAHs, also named heavy chains (HCs), include 13 members (DNAH1-3, 5-12, 14, and 17) in humans (Pazour et al., 2006; Inaba, 2011). In the known axial filament complex, the ODAs play a major role in the beating of cilia and flagella through the ATPase activity of their HCs (Whitfield et al., 2019). Dynein HCs are large proteins that turn the energy of ATP into force supporting the sliding of outer microtubule doublets, which generates the beating of cilia (Pazour et al., 2006). To date, variants in most genes of DNAHs in humans have been reported to be associated with diseases related to cilia or flagella. A common autosomal recessive disorder caused by those variants is PCD, which is characterized by recurrent respiratory tract infections, laterality defects, and/or infertility, with highly genetic and clinical heterogeneity (Fliegauf et al., 2005; Hornef et al., 2006; Pifferi et al., 2010; Li et al., 2016). In the ultrastructure, sperm flagella are similar to cilia, underpinning the common relationship between male infertility with PCD and subfertility in women with PCD due to deficient ciliary function in the oviducts (Lucas et al., 2014). Of interest, variants in *DNAH1* and *DNAH9* genes, reported to be associated with PCD, have been depicted in patients with only male infertility, resulted from asthenozoospermia, without other ciliary disorders (Ben Khelifa et al., 2014; Fassad et al., 2018).

In this study, the proband from family 1 presented with SIT without any other cilia-related symptoms, and compound heterozygous variants c.4109C>T (p.Thr1370Ile) and c.9776C>T (p.Ala3259Val) in the *DNAH17* gene were identified using a combination of WES and Sanger sequencing. The second *DNAH17* compound heterozygous variants, c.612C>G (p.Ile204Met) and c.8764C>T (p.Arg2922Cys), were found in the proband from family 2. The boy presented with dextrocardia and CHD. Cardiac murmurs with cyanosis and recurrent cough were discovered in infancy, suggesting that he may suffer with the cilia-related symptoms. Detailed clinical characteristics of the available family members with *DNAH17* variants are presented in Table 3. It seems that at least 10% (2/20) *DNAH17* compound heterozygous or homozygous carriers have LR asymmetry disorders. There are only a few studies on *DNAH17*, and none was found on studying the gene function due to the large size of DNAH17 molecular mass (510 kDa). In addition, the current knowledge on the crucial part of *DNAH17* playing in flagella destabilization and asthenozoospermia may depend on genetic or environmental factors such as the mutation type, organism or context (Whitfield et al., 2019; Zhang et al., 2020). Actually, different DNAHs members have been shown to play an important role in cilia/flagella formation and cilia/flagella regulation. DNAH1 and DNAH9, homologs of DNAH17, were located to ciliary axonemes and sperm flagella, and responsible for cilia/flagella-related phenotypes (Bartoloni et al., 2002; Olbrich et al., 2002; Ben Khelifa et al., 2014; Fassad et al., 2018). Due to a common highly conserved 9+2 axonemal structure of cilia and sperm flagella, different *DNAH17* mutations may independently cause flagella destabilization, asthenozoospermia or LR asymmetry disorders in specific organelle sharing a common axonemal machinery. Though *DNAH17* expression was detected in testis, brain, lung and other tissues (https://www.ncbi.nlm.nih.gov/gene/8632), the protein was only observed in the flagella by assays of immunoblotting and immunofluorescence staining in various human somatic cell lines, human respiratory epithelial cells and sperm cells (Fagerberg et al., 2014; Whitfield et al., 2019; Zhang et al., 2020). It implies that *DNAH17* expression may be influenced by cell type-specific spatial localization and the switch point in the development of the nodal flow during early embryogenesis. The lack of typical symptoms, such as nasosinusitis and bronchiectasis, in the two patients may be due to absent or low expression of *DNAH17* in specific tissues after the completion of the embryonic development. Biallelic variant types of *DNAH17*, genetic background, and epigenetic modification, as well as environmental factors, may potentially affect the phenotypic manifestation. The possible genotype-phenotype association should be warranted with more *DNAH17*-mutated carriers discovered. Our observation of the potential relationship between *DNAH17* and LR asymmetry disorders may extend the field-of-view for new actor of *DNAH17* in the development of human diseases.

**TABLE 3:** Clinical data of the *DNAH17* variant carriers in different families.

Ped	Case	Sex	Age	GT	Nucleotide change	Amino acid change	Variant type	Infertility	Situs	CHD	References
P1	II:1	M	5 years	CH	c.612C>G, c.8764C>T	p.I204M, p.R2922C	Missense, missense	NA	Dextro	Y	This study
P2	II:1	M	36 years	CH	c.1293_1294del, c.7994_8012del	p.Y431*, p.G2665Efs*4	Nonsense, frameshift	Y	SS	N	Whitfield et al. (2019)
P3	II:1	F	50 years	CH	c.4109C>T, c.9776C>T	p.T1370I, p.A3259V	Missense, missense	N	SIT	N	This study
P4	II:3	M	34 years	CH	c.4445C>T, c.6857C>T	p.A1482V, p.S2286L	Missense, missense	Y	NA	N	Sha et al. (2020b)
P5	II:1	M	32 years	Hom	c.4810C>T	p.R1604C	Missense	Y	SS	N	Zheng et al. (2021)
P6	IV:1	M	43 years	Hom	c.5408G>A	p.C1803Y	Missense	Y	NA	N	Zhang et al. (2020)
	IV:2	M	41 years	Hom	c.5408G>A	p.C1803Y	Missense	Y	NA	N	
	IV:3	M	29 years	Hom	c.5408G>A	p.C1803Y	Missense	Y	NA	N	
	IV:5	F	42 years	Hom	c.5408G>A	p.C1803Y	Missense	N	NA	N	
P7	II:3	M	37 years	Hom	c.5486G>A	p.C1829Y	Missense	Y	SS	N	Whitfield et al. (2019)
	II:4	M	35 years	Hom	c.5486G>A	p.C1829Y	Missense	Y	SS	N	
P8	IV:1	M	39 years	Hom	c.5707C>T	p.R1903C	Missense	Y	NA	N	Zhang et al. (2021) Zhang et al. (2021)
P9	III:2	F	NA	Hom	c.6308C>T, c.11803C>T	p.A2103V, p.Q3935*	Missense, nonsense	N	NA	N	
	IV:1	M	32 years	Hom	c.6308C>T, c.11803C>T	p.A2103V, p.Q3935*	Missense, nonsense	Y	NA	N	
	IV:2	M	42 years	Hom	c.6308C>T, c.11803C>T	p.A2103V, p.Q3935*	Missense, nonsense	Y	NA	N	
	IV:4	M	34 years	Hom	c.6308C>T, c.11803C>T	p.A2103V, p.Q3935*	Missense, nonsense	Y	NA	N	
P10	II:1	M	34 years	CH	c.8512–2A>G, c.13294C>T	NA, p.R4432C	Splicing, missense	Y	NA	NA	Song et al. (2020)
P11	II:4	M	27 years	Hom	c.10496C>T, c.10784T>C	p.P3499L, p.L3595P	Missense, missense	Y	SS	N	Whitfield et al. (2019)
P12	II:1	M	30 years	Het^a^	c.10486_10497dup	p.V3496_P3499dup	Duplication	Y	SS	N	Whitfield et al. (2019)
P13	II:1	M	32 years	CH	c.12915+1G>A, c.13202C>T	NA, p.P4401L	Splicing, missense	Y	NA	NA	Song et al. (2020)

a A variant in the second DNAH17 allele was hypothesized.

DNAH17, dynein axonemal heavy chain 17 gene; Ped, pedigree number; M, male; F, female; GT, genotype; CH, compound heterozygote; Hom, homozygote; Het, heterozygote; N, no; Y, yes; NA, not available; Dextro, dextrocardia; SS, situs solitus; SIT, situs inversus totalis; CHD, congenital heart disease.

Taken together, our research identified compound heterozygous *DNAH17* variants (c.4109C>T and c.9776C>T; c.612C>G and c.8764C>T) in families with LR asymmetry disorders, typical phenotypes of ciliary disorders, including SIT, dextrocardia, and CHD, albeit infertility cannot be excluded. To our knowledge, this is, the first report of relationships between *DNAH17* variants and ciliogenesis, which expands the phenotypic spectrum and benefits genetic counseling. Combined with the reported *DNAH17*-associated asthenozoospermia, we proposed that *DNAH17* compound heterozygous variants, or homozygous variants, may potentially cause a specific disease, the *DNAH17*-associated ciliary/flagellar disorder. The study may be limited by the lack of nasal epithelial brush biopsy samples for ciliary beating and ultrastructure analysis. Further constructing *DNAH17* variant-targeted animal models and performing experimental therapies will facilitate an in-depth comprehension of cellular and molecular mechanisms of ciliary and flagellar defects, and contribute to rectification of the defects.

## Data Availability

The datasets presented in this article are not readily available because the data information is in a controlled state due to the national legislation, specifically the Ministry of Science and Technology of the People’s Republic of China. Data of this project can be accessed after an approval application by the China National GeneBank DataBase (CNGBdb). Please refer to CNGBdb: https://db.cngb.org/, or email: CNGBdb@cngb.org for detailed application guidance. The project accession code CNP0002422 should be included in the application.
